# Confined Fluids in Gel Matrices for the Selective Cleaning of a Tibetan Altar Table

**DOI:** 10.3390/gels11121001

**Published:** 2025-12-11

**Authors:** Chiara Biribicchi, Jessica Chasen, Laura Maccarelli

**Affiliations:** Conservation Center, Los Angeles County Museum of Art, 5905 Wilshire Blvd, Los Angeles, CA 90036, USA; jchasen@lacma.org (J.C.); lmaccarelli@lacma.org (L.M.)

**Keywords:** cleaning, hydrogels, organogels, nanostructured fluids, Tibetan furniture, sustainability, PVA, THM-Py-GC-MS

## Abstract

LACMA’s 19th-century Tibetan Altar Table with Auspicious Symbols is characterized by a complex stratigraphy comprising animal glue-based ground and paint layers, a presumably original tung oil-based varnish, and a dark surface layer composed of a complex mixture of paraffinic wax, shellac, and rapeseed oil, which obscures the object’s original decorative scheme. This study examines the use of nanostructured fluids and organic solvents confined within hydrogels and organogels for the selective removal of the dark surface layer while preserving the underlying paint and varnish. Following the analysis of the artwork’s constituent materials, cleaning tests were conducted and evaluated using visible and ultraviolet fluorescence (UVF) imaging, spectrophotometry, and digital microscopy. The homogeneous absorption of solvent mixtures by the organogels was assessed through gas chromatography–mass spectrometry (GC–MS). Results indicate that confining cleaning fluids within the gels’ porous networks significantly improved solvent retention and control of fluid release. While conventional cleaning methods proved insufficiently selective, the gradual release of a nanostructured fluid containing a small amount of benzyl alcohol, combined with the nanostructural properties of the poly(vinyl alcohol)–sebacic acid (PSA2) hydrogel, enabled targeted removal of the surface layer while preserving the integrity of the underlying layers.

## 1. Introduction

The Los Angeles County Museum of Art (LACMA) has one of the largest collections of Tibetan furniture in the United States, consisting of 48 objects made primarily of painted wood. The collection includes a wide range of forms and sizes—cabinets, tables, and chests—dating roughly from the 17th to 19th centuries. The vast majority of the pieces were acquired between 2005 and 2013 from a single collector, Ruth Sutherlin Hayward, and her family, who sought to place their collection in institutions across the country to deepen the public understanding of Tibetan Buddhism [1].

**Figure 1:**
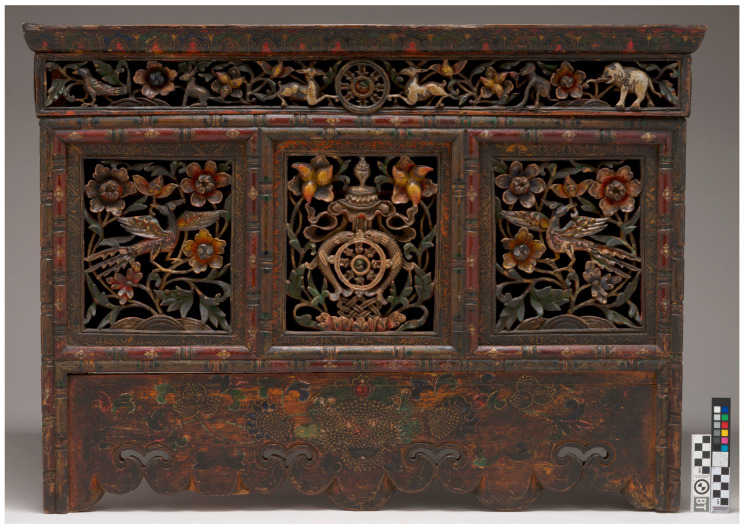
*Altar Table with Auspicious Symbols*, M.2007.109, 19th century, 24 ⅜ × 33 ⅞ × 11 ¾ in, before treatment. Photo Yosi Pozeilov, © Museum Associates/LACMA.

One example from the collection is *Altar Table with Auspicious Symbols* (M.2007.109) (Figure 1). Its construction relies entirely on sophisticated hand-cut wood joinery, with no use of hardware or metal fasteners. In Tibet, where seasonal migration between lower and higher elevations was common, furniture was often designed to break down into smaller components for easier transport [2]. The production of this furniture followed a two-stage process: structural components were first cut, carved, and assembled by woodworkers, after which the pieces were handed to decorative painters, who created incredibly complex painted surface decorations. The painted surfaces were frequently enhanced by raised ground decoration, metal leaf, and elaborate openwork carving. While Tibetan furniture painting techniques and iconography have not received as much scholarly attention as Tibetan *thangka* paintings, it is known that the same pigments, dyes, and binders used in *thangkas* were typically employed in Tibetan furniture decoration as well [3]. On LACMA’s altar table, the central panel features the Eight Auspicious Symbols of Buddhism surrounded by floral and animal motifs. The lower panel depicts a *zipac* or *zeeba*—a mythical, monster-faced creature symbolizing creation, prosperity, and transformation—which seems to reflect Yuan and early Ming art, typical of Tibetan tables [3,4,5]. The table shows a vibrant and relatively intact polychrome surface, protected by a thin, and occasionally disrupted, varnish layer. However, much of this decoration is obscured by a very thick, dark, uneven coating.

Pieces of Tibetan furniture are often found with dark surface layers, commonly attributed to soot from yak butter candles or smoke from fires used for cooking or heating [6,7]. In this case, however, an unidentified coating appears to have been applied to the surface further obscuring its original colors. It has been documented that most Tibetan furniture was destroyed along with the monasteries and temples in which it was kept during the invasion by Chinese troops in 1959 or during the Cultural Revolution (1966–1976) that followed [3]. In order to save cultural materials, monks sometimes moved furniture into storage or transported it out of Tibet. There are reports that pieces were hidden in private homes, where they were exposed to kitchen smoke, or stored in warehouses. Other objects were deliberately coated—often in black or blue household paint—to make their vibrant surfaces unrecognizable, a practice that may also have been applied to the *Altar Table with Auspicious Symbols* [3].

Given that a certain degree of darkening is part of the history of Tibetan furniture, resulting from its functional use in both domestic and monastic or religious contexts, the goal of the cleaning intervention in this study is to reduce, rather than completely remove, the dark material. In the object under examination, while much of the dark layer appears to have been intentionally applied to the surface, there are also believed to be traces of soot. This cleaning approach aims to reveal the colors of the decoration while balancing the preservation of the object’s history with the visibility of its original aesthetic. Partial removal also allows the underlying varnish to be preserved, which, based on the analyses conducted, appears to have been applied when the object was made.

These multiple sources of darkening contribute to the complex stratigraphy observed in LACMA’s case study, as revealed through analysis, making selective cleaning especially challenging with conventional methods. Standard cleaning protocols typically begin with dry techniques—such as polyurethane cosmetic sponges or vulcanized rubber “soot sponges.” This is frequently followed by application of neat solvents—often partially aromatic hydrocarbons, polar solvents such as ketones or alcohols, or, more recently, benzyl alcohol—with cotton swabs. More recently, solvent- and water-based solutions have been delivered to surfaces with thickening agents such as xanthan gum or Pemulen TR-2, or using rigid gels like agarose or gellan [8,9,10,11,12]. While these methods can be effective, they carry risks: some can drive the cleaning solution and/or soiling deeper into the porous stratigraphy, others risk leaving gel residues, and many can undercut the layers which are intended to be retained—i.e., the varnish layer—or soften underlying paint and ground layers due to the high wettability of the treated surface. In addition, the use of petroleum-based solvents is undesirable for human health, safety, and the environment [13]. For these reasons, over the past twenty years, conservators have moved to more tailored cleaning solutions delivered in ways that retain the solution and remove soiling away from the surface [14,15,16,17].

Given the limitations of conventional cleaning systems, LACMA’s Conservation Center tested new products developed within the GREen ENdeavor in Art Restoration (GREENART) project [18,19]. Part of the European Union’s Horizon program, GREENART tackles climate change and supports the UN’s Sustainable Development Goals by creating safe, effective materials and protocols for both preventive and interventive cultural heritage conservation. Within the project, the *Cleaning Systems* working group focuses on developing and testing oil-in-water (O/W) nanostructured fluids, hydrogels, and organogels in combination with greener solvents, with high efficacy and durability, improved safety for conservators, and reduced environmental impact, in line with the European Green Deal [20].

O/W nanostructured fluids are multifunctional amphiphile-based systems mainly composed of water (60–90% *w*/*w*), one or more surfactants, and one or more organic solvents [21]. They contain a small fraction of organic solvents in the form of nanodroplets, which makes it possible to use less solvent in the overall cleaning fluid while increasing the interaction with the target material and facilitating its removal or the swelling, due to the large surface area to volume ratio [22]. The interaction between nanostructured fluids and the material to be removed is significantly different from a classic solubilization process, as it is mostly based on swelling, detaching or dewetting mechanisms, all avoiding the spreading and re-deposition of the solubilized material within the porosity of the substrate [21,23,24].

This selectivity can be further enhanced through the use of innovative hydrogels capable of taking up large amounts of liquid and slowly releasing them onto the treated surface. In recent years, various types of functionalized polymeric networks have been introduced into the field of cultural heritage conservation to help reducing the amount of residues left after treatment and improve the retention capability of traditional gel systems—such as hydroxypropylcellulose, agarose, gellan gum, xanthan gum, and poultices [16,17,22,25,26,27].

These innovative hydrogels possess micro- and nano-scale structural features that form a stable 3D network capable of uniformly loading fluids without disrupting the polymer framework. Their pore structure can be tuned to capture particles of various sizes, and the entire hydrogel can be reused multiple times after rinsing, enhancing sustainability. The hydrogels supplied by the Center for Colloid and Surface Science (CSGI–Florence, Italy) and tested in this study are based on ‘twin-chain’ poly(vinyl alcohol) (PVA) polymer networks, some of which are functionalized with eco-friendly, renewable compounds such as adipic, sebacic, and succinic acid [28,29,30]. Other hydrogels, supplied by the State University of Campinas (UNICAMP—Campinas, Brazil), are derived from sustainable starting materials such as gelatin, alginate, and nanocellulose. Their morphological and physicochemical properties allow the rigidity and retention capacity of the final gel to be adjusted as needed [31].

Another approach to more sustainable cleaning—applied when the use of aqueous solutions is not possible, as in the case of artworks highly sensitive to water—relies on encapsulating organic solvents within organogels [32]. Unlike hydrogels, which have been developed and tested in many fields, including cultural heritage conservation, the application of organogels in this sector and the definition of protocols for their use are still in their early stages. Only a few methods have been explored to date, mostly relying on synthetic materials [32,33,34,35,36,37]. The organogels tested in this study are obtained thorough cost-effective sustainable polyurethane crosslinking and formulated from castor oil (CO), a material with a favorable green chemistry profile thanks to its renewability, biodegradability, and lack of impact on the food chain [33,38,39,40].

Given the complex stratigraphy of the *Altar Table with Auspicious Symbols* and the sensitivity of its varnish and paint layers to free solvents and other traditional cleaning methods, these innovative systems show strong potential for selectively reducing only the unwanted dark surface layer. To this end, following the analysis of the constituent materials to better guide the cleaning, tests were carried out using nanostructured fluids in their free form and confined within hydrogels. The results were then compared with those obtained using benchmarks and pure organic solvents confined within organogels, with the aim of assessing the relative performance of hydrogels and organogels. The study allowed for the selection of the most effective protocol for the *Altar Table with Auspicious Symbols* and to promote the use of innovative cleaning methods that are safer for the environment and for the conservator.

## 2. Results and Discussion

### 2.1. Analysis of the Constituent Materials

In order to inform the subsequent cleaning tests, the relevant components of the painted decoration were analyzed with portable x-ray fluorescence, optical microscopy, scanning electron microscopy coupled with energy dispersive x-ray spectroscopy, and pyrolysis-gas chromatography–mass spectrometry with thermally assisted hydrolysis and methylation. This allowed for a better understanding of the contributions made by specific pigments, minerals, and binders, as well as their respective locations within the paint stratigraphy.

#### 2.1.1. Portable X-Ray Fluorescence (pXRF)

The analyzed spots and the elemental composition detected at each analysis location are presented in Table A1 of the Appendix A.1.

All areas analyzed contain calcium (Ca) and iron (Fe), likely present in the ground layer or possibly embedded in the dark layer on the surface. Barium (Ba) was also frequently detected, suggesting the addition of barium sulphate (BaSO_4_) in the ground layer and the raised decoration or as a filler in combination with other pigments. All areas also contain aluminum (Al), silicon (Si) and traces of titanium (Ti), which suggest the presence of kaolin deposits possibly including Ti [41].

Lead (Pb) and chrome (Cr) are consistently found in orange- and yellow-colored areas, as well as in those where the dark surface layer overlays these paint layers, possibly indicating the use of chrome orange and yellow (PbCrO_4_). Red-colored regions, as well as areas with an underlying red paint layer (i.e., spot 24 in Table A1, Appendix A.1), consistently show the presence of mercury (Hg) and sulfur (S), indicative of the use of cinnabar or vermilion (HgS).

Copper (Cu) and arsenic (As) are frequently co-detected in green-colored regions, sometimes found together with mercury (Hg), which may be associated with the pigment in the underlying red paint layer. The combined presence of Cu and As is consistent with the use of Scheele’s green (AsCuHO_3_) or emerald green (Cu(C_2_H_3_O_2_)•3Cu(AsO_2_)_2_).

The presence of Cu and zinc (Zn) in the gold-colored regions may indicate the use of brass (a Cu-Zn alloy) leaf. Pb is frequently detected in white-painted areas and in combination with other pigments, consistent with the use of lead white (2PbCO_3_·Pb(OH)_2_) both as a standalone pigment and as a component in mixtures, possibly to lighten other colors. No characteristic elemental markers were identified in the blue paint or silver-colored regions.

#### 2.1.2. Optical Microscopy (OM)

**Figure 2:**
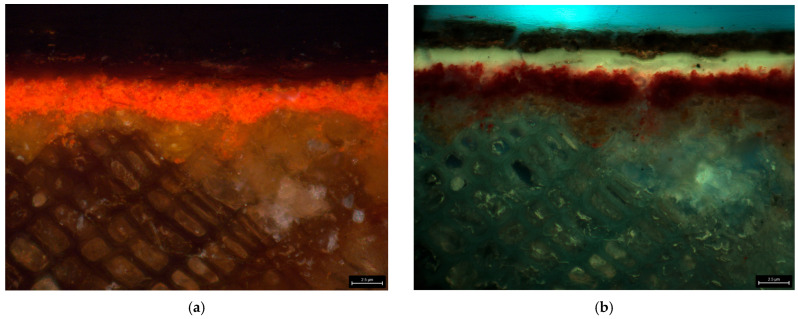
Micrographs of sample XS7 at 400× magnification: (**a**) VIS; (**b**) UVF.

Images taken through OM allowed observation of the stratigraphy of the decorated areas (Figure 2; Table A2, Appendix A.2). They revealed a thin yellow-beige ground layer applied directly onto the wood grain, followed by a thin single—occasionally double—paint layer. An equally thin layer, not readily visible under visible (VIS) light, appears under ultraviolet fluorescence (UVF) with a yellowish fluorescence, indicating the presence of a varnish layer. The absence of soiling at the interface suggests that the varnish was applied shortly after the object was made; for this reason, subsequent cleaning tests aimed at retaining this layer, presumed to be original. Overlying these original layers is a dark coating, which is the target of the cleaning treatment.

#### 2.1.3. Scanning Electron Microscopy Coupled with Energy Dispersive X-Ray Spectroscopy (SEM/EDX) and Polarized Light Microscopy (PLM)

SEM/EDX analysis provided the elemental composition of the different layers. The ground appears to be composed primarily of alumino-silicates, iron (Fe), and potassium (K), possibly kaolinite, with traces of calcium (Ca) and barium (Ba). High levels of Ca were detected only in the surface dark layer, along with traces of Fe, magnesium (Mg), and alumino-silicates.

**Figure 3:**
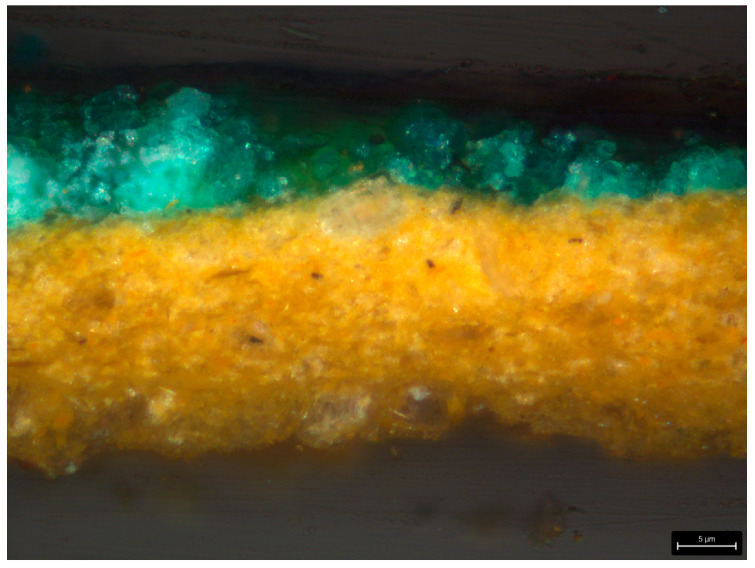
Micrograph of sample XS3 (green paint layer on top of the ground layer) at 400× magnification under VIS light.

The results for the yellow-orange (XS2 and XS4, Table A2, Appendix A.2) and red paint layers (XS1, Table A2, Appendix A.2) are consistent with the XRF analysis, confirming the presence of chrome orange and yellow (PbCrO_4_) and cinnabar or vermilion (HgS), respectively, both also containing barium (Ba) and sulfur (S). The green paint (XS3, Table A2, Appendix A.2; Figure 3) was identified as possibly Scheele’s green (AsCuHO_3_) or emerald green (Cu(C_2_H_3_O_2_)•3Cu(AsO_2_)_2_), with minor amounts of Ca, Ba, and S.

In the silver-colored areas (XS5, Table A2, Appendix A.2), aluminum (Al) leaf was detected, while copper (Cu) and brass (Cu-Zn alloy) foils were identified in the gold-colored decorations (XS6, Table A2, Appendix A.2), likely used to imitate silver and gold, respectively.

**Figure 4:**
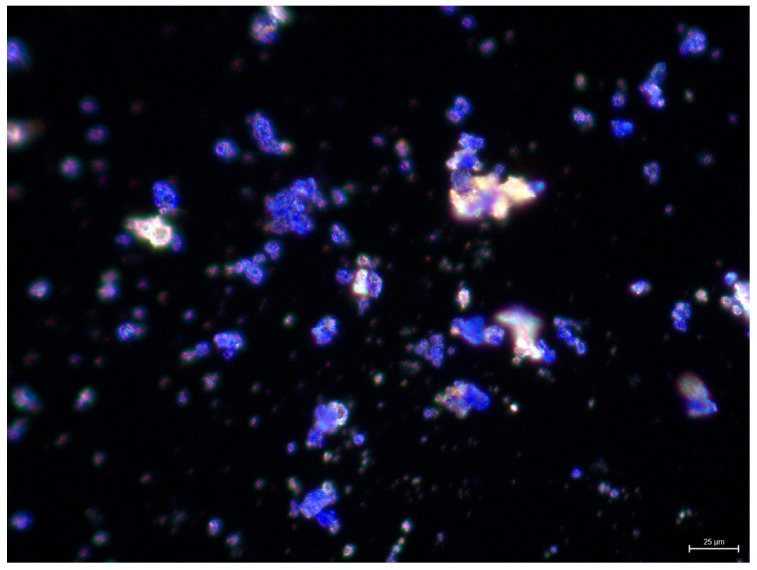
Morphology of ultramarine blue pigment particles observed under polarized light microscopy (PLM) at 400× magnification.

EDX analysis of the blue paint (XS4, Table A2, Appendix A.2) showed sodium (Na), aluminum (Al), sulfur (S), and silicon (Si), with traces of Mg, suggesting the use of ultramarine blue (Na_7_Al_6_Si_6_O_24_S_3_). Following the identification of ultramarine blue via SEM/EDX analysis, additional analyses were undertaken to assess whether the pigment was of synthetic or natural origin. The distinction between natural and synthetic ultramarine blue is primarily determined by the shape and size of the particles. Synthetic ultramarine blue consists of fine, smooth, and uniformly rounded particles, whereas the natural mineral lapis lazuli consists of larger grains with sharper, more irregular edges [42,43,44]. Based on polarized light microscopy (PLM) images, the ultramarine blue used in the Tibetan Altar Table appears to be synthetic (Figure 4).

The pigment palette aligns with what is commonly reported in the literature for pre- and early-20th century Tibetan paints, which often employed mineral-based and synthetic pigments [44,45]. Several studies document the presence of cinnabar (known as *cog-la* or *mtshal-rgod* for the native mineral or *rgya-mtshal* for the synthetic form), synthetic ultramarine blue, chrome yellow, and emerald green in Tibetan murals, paper artifacts, and Thangka paintings [44,45,46,47,48]. A study by J. Mass et al. (2009) also reported the use of Western pigments, including emerald green, ultramarine blue, and chrome yellow, in Tibetan *thangka* paintings from the late 19th and early 20th centuries [49]. Indeed, the trans-regional and cross-cultural trade in Tibetan society likely allowed European synthetic paints to enter soon after their commercial production, especially following the Younghusband Expedition in 1904 and the 1901 Boxer Protocol, which facilitated the import of European goods into Tibet [7].

#### 2.1.4. Pyrolysis-Gas Chromatography–Mass Spectrometry with Thermally Assisted Hydrolysis and Methylation (THM-Py-GC-MS)

Fourier-transform infrared (FTIR) spectroscopy was inconclusive, as the heterogeneous composition of the dark layer did not allow for reliable characterization. For this reason, THM-Py-GC-MS using tetramethylammonium hydroxide was conducted to characterize the organic fraction in the different layers. The thinness of the varnish, paint, and ground layers, along with the possible penetration of the dark layer into the substrate porosity, made the separation of layers and subsequent interpretation difficult.

**Figure 5:**
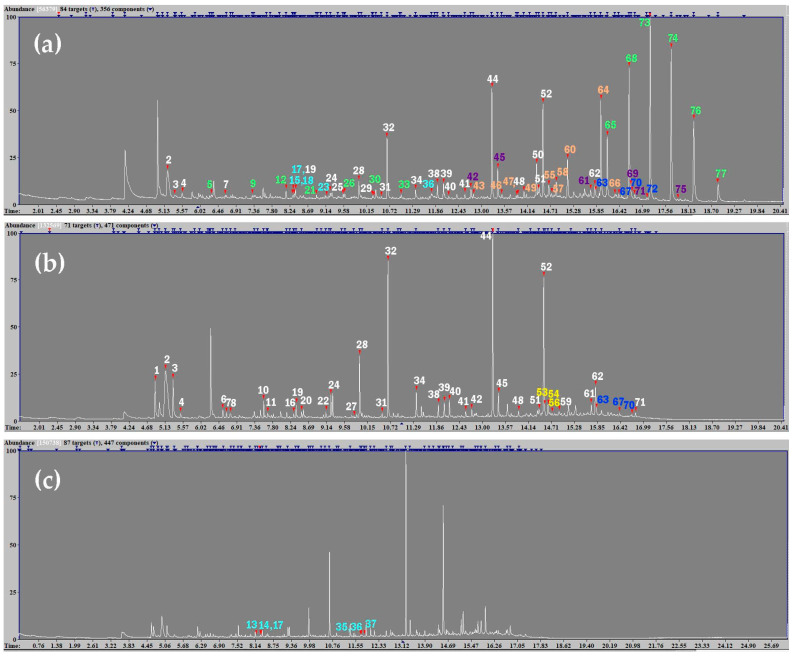
THM-Py-GC-MS chromatogram of the dark surface layer (**a**), the varnish layer (**b**)—likely including paint traces, and the paint layer (**c**)—likely including varnish and ground traces. For each detected material, the main markers are indicated by color: paraffin wax (green), rapeseed oil (purple), shellac (orange), pine resin (dark blue), tung oil (yellow), animal protein (light blue). Additional markers used for material characterization are shown in white. 1: 1,3-dimethoxy-2-propanol; 2: 1,2,3-trimethoxy-propane; 3: 2,3-dimethoxypropan-1-ol; 4: hexanoic acid, methyl ester; 5: 1-decene; 6: 6-heptenoic acid, methyl ester; 7: heptanoic acid, methyl ester; 8: butanedioic acid, dimethyl ester; 9: 1-undecene; 10: octanoic acid, methyl ester; 11: pentanedioic acid, dimethyl ester; 12: 1-dodecene; 13: (1,2-dimethoxyethyl)-benzene; 14: hydroxyproline methylated; 15: blood-unverified 10; 16: 8-nonenoic acid, methyl ester; 17: protein-unverified 8; 18: protein 56-141-156 (glue, egg white, yolk, casein); 19: nonanoic acid, methyl ester; 20: hexanedioic acid, dimethyl ester; 21: 1-tridecene; 22: decanoic acid, methyl ester; 23: blood-unverified 5; 24: heptanedioic acid, dimethyl ester; 25: shellmannose; 26: 1-tetradecene; 27: undecanoic acid, methyl ester; 28: octanedioic acid, dimethyl ester; 29: galactose marker 1; 30: 1-pentadecene; 31: dodecanoic acid, methyl ester; 32: nonanedioic acid, dimethyl ester (azelaic acid, dimethyl ester); 33: 1-hexadecene; 34: decanedioic acid, dimethyl ester; 35: protein 3-blood and glue; 36: protein 1-tofu and blood; 37: diketo-dipyrrole; 38: tetradecanoic acid, methyl ester; 39: undecanedioic acid, dimethyl ester; 40: drying oil-unverified 1; 41: pentadecanoic acid, methyl ester; 42: dodecanedioic acid, dimethyl ester; 43: butolic acid, 6-methoxy, methyl ester; 44: hexadecanoic acid, methyl ester (palmitic acid, methyl ester); 45: tridecanedioic acid, dimethyl ester; 46: 9,10-dimethoxytetradecanoic acid, methyl ester; 47: laccijalaric acid, trimethyl, isomer 2; 48: heptadecanoic acid, methyl ester; 49: laccijalaric acid, trimethyl, isomer 1; 50: laccishellolic acid: dimethyl ester, methyl ether; 51: 9-octadecenoic acid (Z)-, methyl ester; 52: octadecanoic acid, methyl ester (stearic acid, methyl ester); 53: methyl alkylphenyl alkanoate 5; 54: heat bodied oil-unverified 2; 55: 9,10-dimethoxyhexadecanoic acid, methyl ester; 56: heat bodied oil-unverified 1; 57: shellac, unverified 1; 58: jalaric acid, tetramethyl; 59: 9,12-octadecadienoic acid (Z,Z)-, methyl ester (linoleic acid, methyl ester); 60: shellolic acid: dimethyl ester, dimethyl ether; 61: eicosanoic acid, methyl ester; 62: drying oil-unverified 2; 63: methyl dehydroabietate; 64: aleuritic acid, methyl ester, trimethyl ether; 65: tetracosane; 66: aleuritic acid, trimethyl isomers; 67: tetradehydroabietic acid, 7-methoxy-, methyl ester; 68: pentacosane; 69: 13-docosenoic acid, methyl ester; 70: 15-methoxydehydroabietic acid, methyl ester; 71: docosanoic acid, methyl ester; 72: 7-oxodehydroabietic acid, methyl ester; 73: hexacosane; 74: heptacosane; 75: tetracosanoic acid, methyl ester; 76: octacosane; 77: nonacosane.

Analysis of the dark surface layer enabled subtraction of its detected compounds from those identified in the varnish, ground, and paint layers. This layer appears to consist predominantly of paraffin wax, characterized by a series of homologous linear alkanes, containing both even and odd carbon numbers (Figure 5a, Table 1) [50].

All samples containing the dark layer consistently showed the presence of shellac markers (6-methoxy, methyl ester; aleuritic acid derivatives; laccijalaric and jalaric acid derivatives; shellolic and laccichellolic acid derivatives), whereas these compounds were absent or present in traces in samples lacking the layer (Figure 5a, Table 1) [51]. This suggests that shellac, while commonly used as a constituent of varnishes, is more likely incorporated into the dark layer rather than forming part of the varnish. This interpretation is further supported by the yellowish fluorescence of the varnish layer under ultraviolet light (Figure 2b). The observed emission differs from the bright orange fluorescence typical of raw shellac and might at most indicate the use of bleached shellac; however, its marker (chlorine) was not detected in the analyzed samples, indicating that another material was used as the varnish [52].

The dark layer also contains abundant C16 and C18 monocarboxylic fatty acids—palmitic (hexadecanoic) and stearic (octadecanoic) acid methyl esters, respectively—along with glycerol (1,3-dimethoxy-2-propanol; 1,2,3-trimethoxy-propane; 2,3-dimethoxypropan-1-ol), small amounts of short- and medium-chain unsaturated fatty acids, erucic acid (13-docosenoic acid, methyl ester), long-chain fatty alcohols, proteins, traces of carbohydrates, and dicarboxylic acids (Figure 5a, Table 1).

Drying oils can be distinguished from non-drying oils and animal fats based on their content of polyunsaturated fatty acids, which are responsible for the formation of oxidation products that result in the detection of dicarboxylic acids in pyrolyzed samples [53]. Samples containing only the dark surface layer consistently showed high levels of palmitic and stearic acids, while the amounts of dicarboxylic acids and unsaturated hydrocarbons varied with the sampling location, suggesting the possible presence of rapeseed oil and another oil, either within the dark surface layer itself or originating from underlying layers [54].

Rapeseed oil produces dicarboxylic fatty acids, with high amount of C13, C12, and C11, and high-molecular-weight fatty acids (C24, C22:1, C22, C20), with erucic acid (C22:1) being particularly abundant [55]. Its concentration in the samples is estimated at 30–60%, contributing to increased azelaic acid levels. The presence of erucic acid, together with the observed decrease in rapeseed oil content as wax content diminishes, indicates that the oil was likely mixed with wax to produce the dark layer. The levels of palmitic and stearic acids are too high to be attributable solely to rapeseed oil, based on the azelaic/palmitic and palmitic/stearic ratios [55]. The consistent abundance of these acids suggests that the wax layer is intermixed with animal fats, as also supported by the detection of animal proteins in the samples. These compounds are likely associated with soot deposits from burning yak butter candles, a common practice in Tibetan temples [6,7].

Overall, the dark layer appears to be a complex mixture primarily composed of paraffinic wax, rapeseed oil, possibly another drying or semi-drying oil, and shellac together with residues of animal fat. The dark coloration of the layer, together with the absence of characteristic fluorescence emission, is likely attributable to the use of pigments and dyes to intensify its tone, possibly enhanced by a naturally dark shade of shellac.

In samples missing the dark layer, the presence of an oil is more evident based on the higher A/P (0.5) and P/S (~1–1.5) ratios, and the detection of alkylphenyl alkanoates (APAs) which are marker compounds for tung oil (Figure 5b, Table 1) [56]. Historically, Tibetan furniture could be coated with shellac; however, by the late eighteenth century, it was common to finish pieces with a mixture of a drying oil—often tung oil—and resin [3,6,57]. Considering that the object dates from the nineteenth century or later, it is reasonable to conclude that the varnish consists of tung oil alone or tung oil combined with minor amounts of pine resins. Indeed, small quantities of pine resin derivatives (abietic acid methyl esters) were detected in all samples, although it remains unclear whether they originate from the dark surface layer or the varnish (Figure 5b, Table 1).

Paint layer samples and ground layer samples taken from areas where the varnish was absent show protein content consistent with the presence of animal glue, in line with the use of animal glue-based paint and ground layers in Tibetan culture (Figure 5c, Table 1) [58,59].

### 2.2. Cleaning Tests

#### 2.2.1. Standalone Fluids

##### Preliminary Tests on the Back of the Object

Fluids listed in Section 4.2 were first applied on the back of the cabinet for 4-to-6 min using gentle, controlled rolls of a cotton swab across the surface to assess the effectiveness of the cleaning systems before conducting further tests on the decorated areas of the sides and front. Likely due to the thickness and relative insolubility of the layer on the back, it took a minimum of 4 min to see any effect even with the wide range of fluids tested.

Acetone (A), methyl ethyl ketone (MEK), and benzyl alcohol (BA) demonstrated progressively greater ability to solubilize the dark layer while allowing adequate control during application. Furthermore, only slight pigment pickup was observed during the cleaning process at this stage. These preliminary tests revealed the unexpected medium-polar solubility range of the dark, waxy layer, likely due to the presence of shellac and rapeseed oil oxidation products.

Fatty acid methyl esters (FAMEs)—i.e., methyl octanoate (MO), methyl laurate (ML), and methyl soyate (MS)—exhibited limited solubilization ability which was considered advantageous for more controlled, gradual action for more solvent sensitive regions of the table. They were therefore further tested on the decorated areas.

Several nanostructured fluids—namely the GREENART formulations containing benzyl alcohol (NBA) or methyl tetrahydrofuran (NWX), the Nanorestore Cleaning^®^ Apolar Coating (NAC), and Nanorestore Cleaning^®^ Polar Coating G (NPG)—showed effectiveness in reducing the black layer and were therefore carried over into additional testing phases. The other nanostructured fluids tested—i.e., those containing diethyl ketone (NDEK), diethyl carbonate (NDEC), and butyl acetate (NBuA)—proved ineffective as neat fluids and were excluded from further testing. However, it is important to note that nanostructured fluids are specifically designed for use in combination with gel matrices or other retentive systems which allow for longer contact times. As such, the performance observed in their liquid state in short tests may not accurately represent their full cleaning potential when longer applications are carried out. Indeed, further tests carried out by confining the nanostructured fluids in Nanorestore Gel^®^ Peggy 6—used as a reference material that has been already extensively studied—demonstrated that the use of a retentive system significantly enhances the dissolution ability of all the nanostructured fluids.

##### Tests on the Decorated Side Panel of the Object

The fluids that demonstrated partial or complete removal of the dark layer on the back—i.e., A, MEK, BA, MO, ML, MS, NBA, NWX, NAC, and NPG—were tested on the decorated side panel, which is more representative of the object’s presentation surfaces. In these areas, a thin varnish layer, not present on the back, coats the paint layer, while the dark surface layer appears significantly thinner and unevenly distributed, likely as a result of previous cleaning treatments. For these reasons, more nuanced outcomes were observed, with the variations attributable to the sensitivity of the varnish and paint layers to the applied cleaning systems.

**Figure 6:**
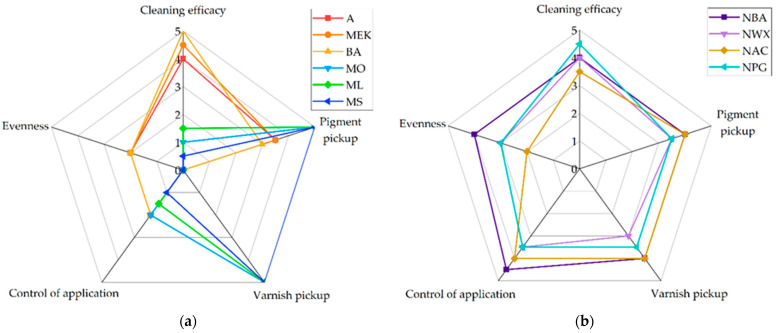
Radar diagrams showing the results of the cleaning tests carried out on the decorated side of the object with organic solvents (**a**) and the selected nanostructured fluids (**b**). NBA is an aqueous nanostructured fluid containing the solvent benzyl alcohol (BA), NWX is an aqueous nanostructured fluid containing the solvent methyl-tetrahydrofuran (m-THF), NAC stands for Nanorestore Cleaning^®^ Apolar Coating, NPG stands for Nanorestore Cleaning^®^ Polar Coating G, A is acetone, MEK is methyl ethyl ketone, BA is benzyl alcohol, MO is methyl octanoate, ML is methyl laurate, MS is methyl soyate. Cleaning systems details are specified in Materials and Methods (Table 3).

Tests were performed using a fixed application interval for each fluid—i.e., 2 min—extended up to 6 min when no dissolution was observed. The results are presented in the radar diagram shown in Figure 6, which evaluate cleaning efficacy, varnish pickup, pigment pickup, application control, and evenness on a scale from 0 to 5.

High cleaning efficacy was observed in the areas treated with A, MEK, and BA; however, all three also resulted in the removal of the varnish layer. In contrast, FAMEs showed limited ability to solubilize the dark surface layer but preserved the integrity of both the varnish and the underlying paint, suggesting that lower polarity may be more suitable for preserving the original materials during a selective cleaning treatment.

**Figure 7:**
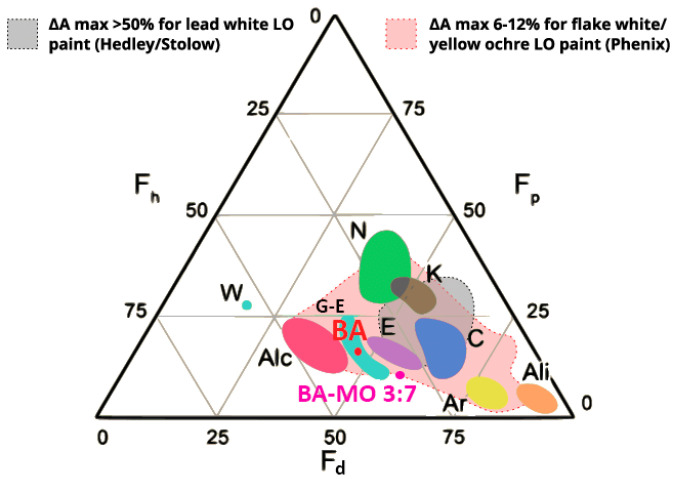
The Teas chart, including the ‘peak swelling’ region identified by Hedley [63] in grey, and the extended ‘peak swelling’ region based on different paint types identified by Phenix in light red. BA = benzyl alcohol, BA-MO 3:7 = benzyl alcohol and methyl octanoate mixture at the defined 3:7 *v/v* ratio, W = water, N = nitrogen containing solvents, K = ketones, Alc = alcohols, G-E = glycol ethers and esters, E = esters, C = chlorinated solvents, Ar = aromatics, Ali = aliphatics’ LO = linseed oil. Image adapted from Baij et al. [66], Chelazzi et al. [17], and Phenix [65].

For this reason, at this stage, a solvent mixture was introduced to reduce both the polarity and cleaning strength of BA. This proved to be the most effective among the tested organic solvents. To this end, mixtures of BA and MO were tested at different relative concentrations. MO was selected as the low-polar organic solvents added to BA to reduce polarity, due to the greener chemical profile of FAMEs compared to petroleum-based solvents [60,61,62]. Among the various FAMEs, MO was specifically selected based on its loading time in organogels and its physical properties, which are more closely aligned with those of BA. The swelling behavior and loading time of organogels in organic solvents were studied by CSGI. Cleaning tests were carried out to define the minimum concentration of BA required for effective solubilization, resulting in a formulation consisting of 70% MO and 30% BA. This mixture shifts toward lower polarity and lies outside the high-swelling region of oils (>50%) defined by Hedley and Stolow (1980) and sits at the edge of the broader swelling range reported by Phenix (2002), between 6% and 12%, making it safer for the tung oil-based varnish (Figure 7) [63,64,65].

The nanostructured fluids provided more balanced outcomes, better combining the goal of uniformly reducing the dark surface layer with the need to minimize interaction with the underlying layers. Among the nanostructured fluids tested on the decorated areas, NBA performed the best overall. For NWX, NAC, and NPG, high cleaning efficacy was usually counteracted by unevenness, reduced application control, or slight varnish or pigment pickup.

#### 2.2.2. Analysis of the Organogel’s Solvent Uptake

After reducing the polarity and cleaning strength of BA by formulating the BA-MO 3:7 *v*/*v* mixture, it was important to evaluate the possibility of incorporating this mixture into an organogel, aiming to achieve a more gradual and selective cleaning treatment. To this end, it was necessary to verify the extent to which the organogel absorbed the two solvents in the mixture and to ensure that both were absorbed at the defined concentrations.

**Figure 8:**
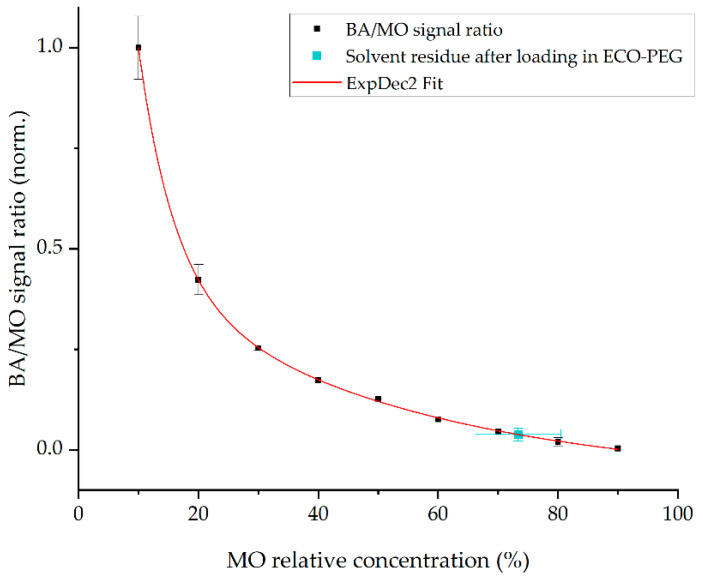
Calibration curve built based on the BA/MO signal ratio at different relative concentrations, showing the relative concentration of the solvent residue analyzed after loading the solvent mixture in the organogel ECOP.

The data used to generate the calibration curve indicate that the variability of the MO/BA ratio increases with higher relative concentrations of MO, resulting in a larger margin of error (Figure 8). Measurements of the residual solution remaining inside the vials after adsorption by the organogel are generally consistent with the initially defined composition of the solvent mixture, showing MO concentrations ranging from ~66% to 81%. This suggests that the solvent mixture is taken up relatively uniformly by the organogel. However, a certain degree of variability should be considered due to the small volume of solvent used for the uptake tests.

#### 2.2.3. Final Testing

Preliminary tests with the solvents in their liquid state allowed for the identification of the nanostructured fluids and organic solvents best suited for the cleaning target. The final testing phase aimed to evaluate the effectiveness and control of the cleaning treatment when the fluid is confined in a retentive system, by comparing the performance of the available hydrogels and organogels (Table 2). Final tests were carried out on the lower portion of the object’s front, specifically on the orange-colored areas. The gel systems were examined in terms of conformation to the surface, controlled release, and general cleaning result.

NBA—the O/W nanostructured fluid based on benzyl alcohol—was the only formulation that allowed for the gradual removal of the dark surface layer without affecting the underlying varnish or paint layers. Therefore, it was identified as the most effective and selective cleaning fluid and tested in combination with the PVA-based hydrogels developed by the CSGI (Nanorestore Gel^®^ Peggy 6, PP3, PSA2, PSU2, PAD) and the nanocellulose-alginate (CNF-A) and nanocellulose-gelatin (CNF-G) hydrogels developed by UNICAMP.

In parallel, the organogels developed by the CSGI (ECO, ECOH, ECOP) loaded with a solvent mixture of BA and MO in a 3:7 (*v*/*v*) ratio were tested. This allowed for a comparison between two different cleaning methods—i.e., nanostructured fluids loaded in hydrogels and solvent mixtures confined into organogels—both seeking to enable sufficient swelling of the dark surface layer to facilitate its mechanical removal with cotton swabs, while minimizing interaction with the underlying paint and varnish layers.

Established benchmarks were used for comparison with the selected cleaning systems—i.e., Nanorestore Gel^®^ Peggy 6, Pemulen™ TR-2, and Evolon^®^ CR—and a more recently developed hydrogel, the XKA hydrogel described in Section 4.2 of Materials and Methods.

**Figure 9:**
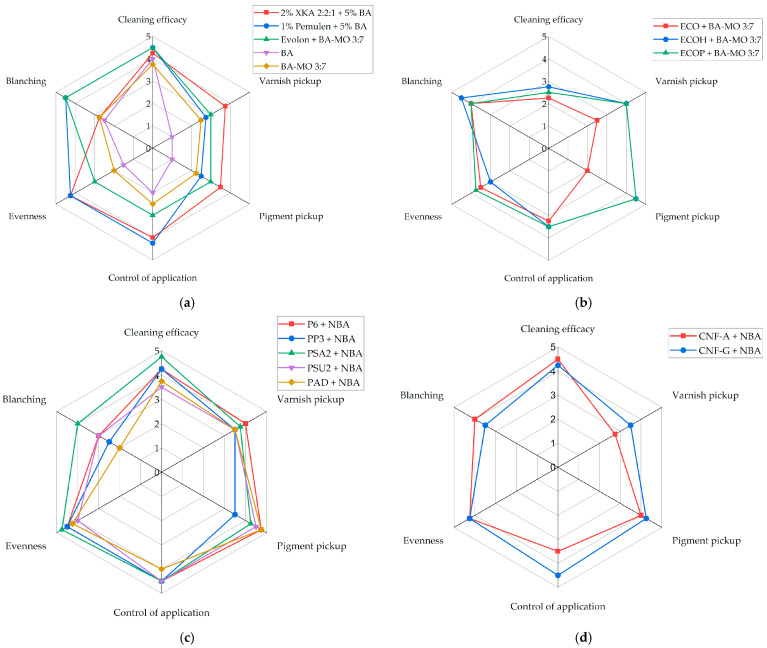
Radar diagrams presenting the results of the final cleaning tests on the decorated area of the object’s front, comparing the benchmarks (**a**), the solvent mixture confined in organogels (**b**), the selected nanostructured fluid confined in hydrogels provided by CSGI (**c**), and the selected nanostructured fluid confined in hydrogels provided by UNICAMP (**d**).

All cleaning systems were applied for the timeframes specified in Table 2. Water-based systems were rinsed using pH 6.5, 6000 microSiemen-adjusted water (AW), delivered either in the same gel used for treatment or, in the case of Pemulen TR-2, with a cotton swab. The results are presented in the radar diagrams shown in Figure 9, which evaluate cleaning efficacy, varnish pickup, pigment pickup, application control, and evenness on a scale from 0 to 5. The evaluation is based on visual inspection as well as technical analyses, including visible (VIS) and ultraviolet fluorescence (UVF) photography, spectrophotometry, and digital microscopy.

**Table 2:** Cleaning systems selected for final testing.

ID	Fluid	Gel	Cleaning System	Application Time
1	NBA	Peggy 6	Nanostructured fluid + hydrogel	8′ (2′ intervals)
2	NBA	PP3	Nanostructured fluid + hydrogel	10′ (2′ intervals)
3	NBA	PSA2	Nanostructured fluid + hydrogel	8′ (2′ intervals)
4	NBA	PSU2	Nanostructured fluid + hydrogel	8′ (2′ intervals)
5	NBA	PAD	Nanostructured fluid + hydrogel	8′ (2′ intervals)
6	NBA	CNF-A	Nanostructured fluid + hydrogel	8′ (2′ intervals)
7	NBA	CNF-G	Nanostructured fluid + hydrogel	10′ (2′ intervals)
8	DI water + 5% BA	2% XKA 2:2:1	Water and solvent + hydrogel	4′ (2′ intervals)
9	pH 6.5-AW + 5% BA	1% Pemulen™ TR-2	Water and solvent + emulsifier	1′ 15″
10	BA-MO 3:7	ECO	Solvent mixture + organogel	8′ (2′ intervals)
11	BA-MO 3:7	ECOH	Solvent mixture + organogel	8′ (2′ intervals)
12	BA-MO 3:7	ECOP	Solvent mixture + organogel	8′ (2′ intervals)
13	BA-MO 3:7	Evolon^®^	Solvent mixture + microfilament textile	4′ (2′ intervals)
14	BA	-	Organic solvent	1′ 15″
15	BA-MO 3:7	-	Organic solvent mixture	2′ 45″

Ultraviolet fluorescence (UVF) imaging was used to assess possible thinning or removal of the varnish layer, based on its fluorescence emission (Table A4, Appendix A.4). Noticeable varnish and pigment loss were observed after treatment with BA, while partial thinning or localized varnish removal was also evident in areas treated with Pemulen TR-2, as well as with the XKA-based system, ECO organogel, and Evolon loaded with the BA–MO mixture. This result is possibly related to the higher fluid release of Pemulen TR-2, Evolon, and ECO compared to other hydrogel and organogel systems. At the same time, ECO showed insufficient removal of the dark layer, as did ECOP, ECOH, and—although to a lesser extent—PP3. Their higher rigidity made it difficult for them to fully conform to rough surfaces, resulting in uneven and overall unsatisfactory removal of the dark layer. Differences in gels adaptability to the rough surface morphology, and the resulting patchy removal, are visible in digital microscopy images (spots 2, 10, 11, 12 in Table A5, Appendix A.5). Uneven fluid distribution caused prolonged contact in certain areas, leading to over-cleaning only in those regions.

The remaining gel-based systems generally preserved the integrity of the varnish layer. CNF-A showed a stronger, less controlled cleaning action compared to the other hydrogels, likely due to its faster fluid release, which led to partial varnish and pigment removal. PSU2 and PAD provided gradual, controlled cleaning but did not adequately remove the dark layer, which caused slight blanching of the treated area. CNF-G produced a more uniform surface, while PSA2 provided the most controlled cleaning, with no detectable varnish or pigment loss.

**Figure 10:**
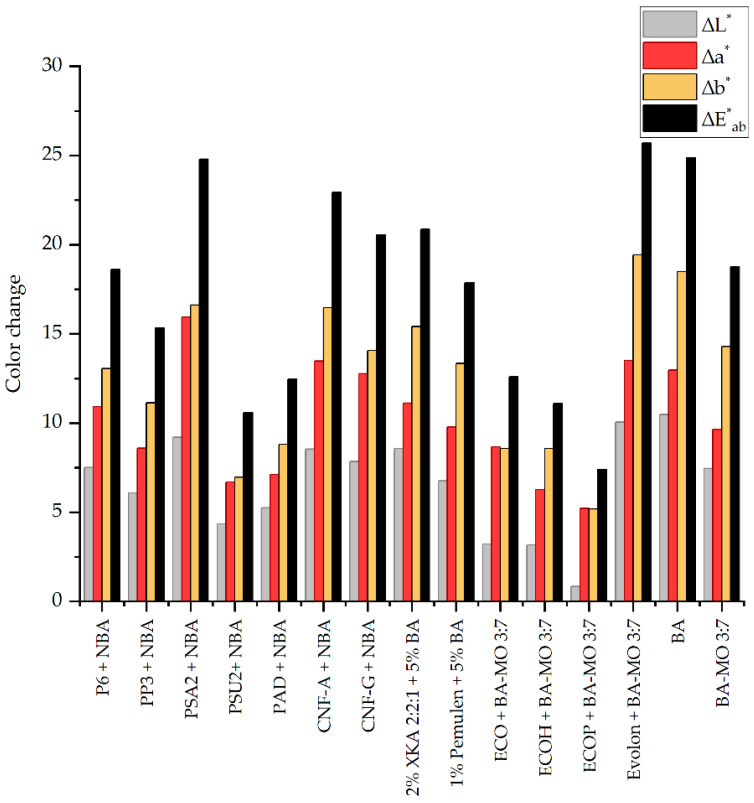
Colorimetric evaluation: changes in L*, a*, and b* values before and after treatment.

Colorimetric data are consistent with the visual and microscopic observations, indicating more aggressive and less controlled action of solvents when not confined within organogel systems (Figure 10). Furthermore, the highest ${∆E}_{ab}^{*}$ values were recorded in areas treated with BA (14), Evolon loaded with BA–MO 3:7 *v*/*v* (13), and PSA2 + NBA (3). The first two treatments showed a marked increase in the *b component, indicating a stronger shift toward yellow, consistent with varnish and pigment removal. In contrast, PSA2 produced a greater shift toward red (*a component), suggesting the effective removal of the surface layer and preservation of the underlying paint.

Among the systems tested, PSA2 provided the most even and effective cleaning, providing visually uniform results well-suited to the surface’s sensitivity and the conservation objectives (Figure 9; and spot 3 in Table A5, Appendix A.5).

## 3. Conclusions

The analysis of the constituent materials of LACMA’s *Altar Table with Auspicious Symbols* (M.2007.109) placed the work in the 19th century or later and informed the definition of the appropriate conservation goals and treatment strategies.

Elemental analyses (pXRF, SEM/EDX) revealed a pigment palette composed of mineral-based pigments and metallic decorations imitating silver and gold, traditionally used in pre-20th-century Tibetan paintings and furniture (cinnabar/vermilion, brass, copper, aluminum-based metallic decorations) [7,44,45]. They also showed the presence of synthetic pigments developed in the 19th century (emerald green, chrome yellow and orange, and possibly synthetic ultramarine blue), which became increasingly more common in Tibet from the late 19th to the early 20th century [7,49]. Lead chromate (chrome yellow) was first synthesized in 1809, with commercial production beginning between 1814 and 1816 in Europe; it became widely used as a pigment in the second quarter of the 19th century [67,68]. Emerald green was first formulated in Germany in 1814 and spread to East Asia from the 1850s onwards, while synthetic ultramarine blue was developed in 1828 [69,70].

THM-Py-GC-MS provided detailed characterization of the organic components, shedding light on the complex stratigraphy. Although samples were collected trying to isolate individual layers, inevitably led to contamination from adjacent layers, complicating interpretation. Taking possible contamination into account, the stratigraphy appears to consist of: (1) an animal glue-based ground layer applied directly to the wood; (2) a paint layer bound with animal glue; (3) an original varnish composed of tung oil and, possibly, a minor portion of pine resin; (4) a dark layer covering the decorative scheme that appears to be a later addition, consisting of paraffin wax likely mixed with rapeseed oil, shellac, possibly pine resins and dyes or pigments producing the dark color, and animal fats from soot deposits presumably produced by yak butter candles.

Stratigraphic data guided the design of cleaning tests, developed to reduce the dark surface layer only, based on the polarity and sensitivity of the layers to be removed or preserved. The varnish and paint layers proved sensitive to the tested benchmarks, while the innovative materials developed within the GREENART project proved more suitable to achieve selective removal of the dark layer while preserving the underlying paint and varnish.

To varying degrees, both hydrogels and organogels demonstrated increased control of application, enabling more gradual and safer removal of the dark layer. Organogels (ECO, ECOP, ECOH) allowed for a slow release of the selected solvent mixture (BA-MO 3:7 *v*/*v*) but resulted in uneven cleaning, proving less suited to the rough surfaces. Hydrogels loaded with the selected benzyl alcohol-based nanostructured fluid (NBA) provided greater control, adaptability to the surface, more uniform results, and preservation of the underlying layers. The PSA2 hydrogel, loaded with NBA, produced the most satisfactory outcomes and was adopted as a greener, safer cleaning system for the treatment of the *Altar Table with Auspicious Symbols*.

## 4. Materials and Methods

### 4.1. Analysis of the Constituent Materials

#### 4.1.1. Portable X-Ray Fluorescence

Thirty-one spots were analyzed with a Bruker Tracer 5 pXRF spectrometer (Brucker, San Jose, CA, USA), using a 3 mm collimator, and an Olympus Innov-X Delta DP-2000 (Innov-X Systems Inc., Woburn, MA, USA) in an air atmosphere. The Olympus spectrometer was used in Alloy Plus, Precious Metal, and Geochem modes, which use predefined parameter routines to provide alloy identification, perform the accurate elemental analysis of precious metals, and conduct geochemical analysis, respectively. The Bruker spectrometer was used in Spectrometer mode. X-ray tube parameters were set at 40 kV/100 μA and 35 s acquisition.

#### 4.1.2. Optical Microscopy (OM)

Seven cross-sections (Table A2, Appendix A.2) were embedded in Technovit 2000 LC resin (Kulzer Technik, Wehrheim, Germany), cured in a Technotray POWER light polymerization unit (Kulzer Technik, Wehrheim, Germany), and examined using Optical Microscopy with a DMC4500 Digital Microscope Camera (Leica Microsystems, Boston, MA, USA) under VIS and UVF at 160× and 400× magnification.

#### 4.1.3. Scanning Electron Microscopy Coupled with Energy Dispersive X-Ray Spectroscopy (SEM/EDX)

SEM/EDX analyses were carried out with a FlexSEM 1000 Scanning Electron Microscope (Hitachi High-Tech America Inc., Chatsworth, CA, USA) coupled with a Nano EDX system (Bruker, Berlin, Germany). Variable pressure mode (30 Pa), backscattered electron detection mode (BSE), and 20 kV voltage were used.

#### 4.1.4. Polarized Light Microscopy (PLM)

Scrapings of the blue pigment were observed under polarized light microscopy (PLM) to determine whether synthetic or natural ultramarine blue was used. The analysis was carried out with an Optical Microscopy with a DMC4500 Digital Microscope Camera under VIS light at 400× magnification.

#### 4.1.5. Pyrolysis-Gas Chromatography–Mass Spectrometry with Thermally Assisted Hydrolysis and Methylation (THM-Py-GC-MS)

Pyrolysis-gas chromatography–mass spectrometry with thermally assisted hydrolysis and methylation using tetramethylammonium hydroxide (THM-Py-GC-MS) was used to complement the information obtained with FT-IR and to further characterize the organic compounds in the stratigraphy. Analyses were performed on a Frontier PY-3030D multi-shot pyrolyzer (Frontier Laboratories Ltd., Fukushima, Japan) interfaced to an Agilent 8890 Gas Chromatograph (GC) System with a 5977C inert Mass Selective Detector (MSD) (Agilent Technologies, Little Falls, DE, USA). Samples were placed into 50 µL stainless steel Eco-cups and treated with 2.5 µL of 25% tetramethylammonium hydroxide (TMAH) in methanol, then pyrolyzed at 550 °C. A J&W DB-5MS capillary column (20 m  ×  0.18 mm  ×  0.185 µm; Agilent Technologies, Santa Clara, CA, USA) attached to a Vent-Free adaptor (Frontier Laboratories, Fukushima, Japan) was used, resulting in a 26 m effective column length, with the helium flow set to 0.7 mL per minute. The split ratio was set at 50:1. The GC oven was programmed as follows: 35 °C with a three-minute hold; ramp to 160 °C at 20 °C per minute; then to 280 °C at 15 °C per minute; followed by a ramp to 315 °C at 5 °C per minute and a final two-minute isothermal hold. The mass range was set between 12.00 and 600.00, with a 150 threshold, and 3.123 u/s. Samples were collected from the orange, green, and red paint layers, as well as from the varnish layer and the overlying black layer, trying to isolate each layer to avoid peak interference.

AMDIS (Automated Mass Spectral Deconvolution and Identification System) was used for data processing, using the retention index (RI) as a sorting parameter and the library developed in the Getty research, setting 80% as the minimum match factor [71,72]. Data interpretation was conducted using ESCAPE (Expert System for Characterization using AMDIS Plus Excel), an expert system developed by researchers at the Getty Conservation Institute (GCI) that uses an Excel report template to sort marker compounds into material categories [73].

### 4.2. Cleaning Systems

Selected nanostructured fluids and organic solvents were tested in their liquid state with cotton swabs to evaluate the solubility range of the dark layer, as well as that of the varnish and paint layers to be preserved (Table 3).

The best-performing fluids were confined in either the hydrogels or organogels, listed in Table 4 based on their compatibility with the gels. Hydrogels and organogels were provided by State University of Campinas (UNICAMP—Campinas, Brazil) and the Center for Colloid and Surface Science (CSGI—Florence, Italy) within the GREENART project (GREeen Endeavor in Art ResToration) [18].

A hydrogel prepared in the LACMA conservation studio, referred to as XKA, was also tested as a simple and easy-to-prepare alternative to the hydrogel described in Table 4. The XKA hydrogel was formulated as a 2 wt% solution consisting of a 2:2:1 mixture of xanthan gum (Spectrum chemical, Gardena, CA, USA), konjac (Modernist Pantry, Eliot, ME, USA), and agarose (Cole-Parmer, Vernon Hills, IL, USA) in deionized (DI) water. The mixture was stirred using a magnetic stirrer and heated in a water bath to 90 °C while stirring continuously. Upon reaching the target temperature, 5 wt% of BA was added to the mixture under constant stirring. The resulting gel was cast, allowed to cool to room temperature, and then stored in a sealed container to minimize solvent evaporation.

Pemulen™ TR-2 (MuseuM Services Corporation, South St Paul, MN, USA) and Evolon^®^ (Blick, Galesburg, IL, USA) were used as benchmarks for comparison with the tested cleaning systems. Pemulen™ was prepared as a 1 wt% aqueous solution, adjusted to pH 6.5 with triethanolamine (TEA), and including 5 wt% BA.

### 4.3. Cleaning Tests

#### 4.3.1. Standalone Fluids

Tests were performed using a fixed application time for each fluid—4 min on the back of the object and 2 min on the decorated areas. The application ceased earlier if signs of paint or varnish removal appeared or extended up to 6 min when no dissolution was observed. Deionized water was used for the clearance step after testing the nanostructured fluids.

The results of the cleaning tests on the decorated areas of the object were evaluated using a radar diagram including cleaning efficacy, varnish pickup, pigment pickup, application control, and evenness. Each parameter was quantitatively evaluated on a scale from 0 to 5, with 0 indicating complete non-fulfillment and 5 indicating full fulfillment of the defined performance criteria.

#### 4.3.2. Final Testing

The treated areas were analyzed before and after treatment using technical photography in visible (VIS) light and ultraviolet fluorescence (UVF), spectrophotometry, and digital microscopy, to assess cleaning efficacy, evenness, and any pigment or varnish pickup. The results were used to build a radar diagram including the same parameters outlined in Section 4.3.1 for the decorated areas.

VIS images were taken using a GFX 100 camera (Fujifilm, Tokyo, Japan) equipped with Fujifilm GF 63 mm F2.8 lens with Hoya EVO UV(0) filter, and Xenon Flash Strobe lights. UV induced VIS fluorescence (UVF) images were acquired with a Fujifilm GFX 100 camera equipped with Fujifilm GF 63 mm F2.8 lens with Hoya EVO UV(0) filter and Peca #916 Filter, and a Waveform Lighting RealUV 365 nm LED radiation source with MidOpt BP365 Filter (x4). The examination of the surface morphology was carried out using the digital microscope HRX-01 (Hirox Co., Oradell, NJ, USA) (magnification 100×) under visible light (VIS).

A CM-2600d spectrophotometer (Konica Minolta Sensing Inc., Ramsey, NJ, USA) with a 1 cm aperture was used. Data were acquired using the OnColor Software v6.3.6.0 with a specular component excluded (SCE), then elaborated by using the CIELAB ΔE*_ab_ (Equation (1)) [75].

(1) $${∆E}_{ab}^{*}=\sqrt{{\left({∆L}^{*}\right)}^{2}+{\left({∆a}^{*}\right)}^{2}+{\left({∆b}^{*}\right)}^{2}}$$

where ΔE*_ab_ represents the total color difference, ΔL* is the difference in lightness, Δa* is the difference on the red/green axis, and Δb* is the difference on the yellow/blue axis. Three measurements for each area were performed to reduce uncertainty.

#### 4.3.3. Analysis of the Organogel’s Solvent Uptake

To ensure that the organogels effectively absorbed the benzyl alcohol and methyl octanoate mixture at the defined 3:7 *v*/*v* ratio, gas chromatography–mass spectrometry (GC-MS) analyses were performed.

A calibration curve was generated by analyzing benzyl alcohol and methyl octanoate mixtures at the following volume ratios: 1:9, 2:8, 3:7, 2:3, 1:1, 3:2, 7:3, 8:2, and 9:1. Each mixture was analyzed in triplicate and the integral signal used to calculate relative proportions between BA and MO. Mean and standard deviation were calculated and used to build the calibration curve with OriginPro 2025 using a two-phase exponential decay function (Table A3, Appendix A.3).

Once the calibration curve was built, five vials were prepared by loading ECOP in a 3:7 (*v*/*v*) mixture of BA and MO 2.5 h. Five pieces of the ECOP, each weighing 0.3 ± 0.02 g, were individually placed in vials containing 2.0 ± 0.03 g of solvent mixture. The samples were allowed to absorb the fluid for 2.5 h, based on the swelling time of the organogel in the selected solvents. The amount of liquid adsorbed by the organogel was calculated, and the remaining solution in each vial was analyzed using the same protocol as the calibration curve to determine whether the solution was adsorbed uniformly, without altering the original ratio.

The same GC/MSD system and column described in Section 4.1.5 were used, equipped with an Agilent 7693A automatic liquid sampler (ALS) (Agilent Technologies, Little Falls, DE, USA). The injection volume was set to 0.1 µL with an L1 air gap of 0.2 µL. Methanol HPLC grade (CAS n. 67-56-1; Fisher Scientific, Carlsbad, CA, USA) was used as wash solvent A and toluene ACS 99.5% (CAS n. 108-88-3; Alfa Aesar, Ward Hill, MA, USA) as wash solvent B. For both solvents, three pre-injection washes were performed using the maximum wash volume (8 µL), while no post-injection washes were applied. The sample wash settings included nine washes at the maximum volume (8 µL), with six sample pumps. A variable plunger speed was selected. The draw and dispense speeds for both solvent and sample washes were set at 300 µL/min and 6000 µL/min, respectively, while the injection speed was set at 500 µL/min.

The GC oven was programmed as follows: 35 °C with a 1.5 min hold; ramp to 160 °C at 15 °C per minute; then to 300 °C at 20 °C per minute and a final fifteen-minute isothermal hold. The split ratio was set at 150:1.

## Figures and Tables

**Table 1:** Main markers for each detected material, as shown in Figure 5.

Material	Main Markers	Main Marker Compounds
Animal fat	Monocarboxylic fatty acid methyl esters	44 *, 48, 52 *
	Miscellaneous	15, 17, 18, 23, 29, 36
Paraffin wax	Linear hydrocarbons	5, 9, 12, 21, 26, 30, 33, 65, 68, 73, 74, 76, 77
Rapeseed oil	Glycerol	1, 2, 3
	Monocarboxylic fatty acid methyl esters	61, 69, 71, 75 *
	Dicarboxylic fatty acid methyl esters	39, 42, 45 *
Shellac	Monocarboxylic fatty acid methyl esters	43, 46, 55
	Sesquiterpenic acid derivatives	47, 49, 50, 58, 60
	Aleuritic acid derivatives	64, 66
	Miscellaneous	57
Pine resin	Abietic acid methyl esters	63, 67, 70, 72
Tung oil	Glycerol	1, 2, 3
	Monocarboxylic fatty acid methyl esters	44 *, 52 *, 59
	Dicarboxylic fatty acid methyl esters	32 *
	Alkylphenyl alkanoates	53, 54, 56
Animal glue	Amino acids	14
	Pyrroles	37
	Miscellaneous	13, 15, 17, 18, 23, 29, 35, 36

* Interpretation was based not only on marker identification but also on the analysis of the relative ratios of azelaic to palmitic (A/P) and palmitic to stearic (P/S) fatty acid methyl esters, which allow the distinction between different oils, proteinaceous materials, and fats.

**Table 3:** Nanostructured fluids and organic solvents used in the tests. OS: organic solvent.

Name	ID	Category	CAS	Provider
Acetone	A	OS	67-64-1	Thermo Scientific (Waltham, MA, USA)
Methyl ethyl ketone	MEK	OS	78-93-3	Conservation Support Systems (Santa Barbara, CA, USA)
Benzyl alcohol	BA	OS	100-51-6	Thermo Scientific
Shellsol A100	SA100	OS	64742-95-6	Conservation Support Systems
Methyl octanoate	MO	OS	0111-11-5	Tokyo Chemical Industry (Tokyo, Japan)
Methyl laurate	ML	OS	111-82-0	Thermo Scientific
Methyl soyate (SOYGOLD 1100)	MS	OS	67784-80-9	ChemPoint (Bellevue, WA, USA)
HCO CeXOH-DEK	NDEK *	Nanostructured fluid	-	CSGI
HCO CeXOH-DEC	NDEC *	Nanostructured fluid	-	CSGI
HCO CeXOH-BuAco	NBuA *	Nanostructured fluid	-	CSGI
HCO WX	NWX *	Nanostructured fluid	-	CSGI
HCO BZ2	NBA *	Nanostructured fluid	-	CSGI
Nanorestore Cleaning^®^ Apolar Coating	NAC	Nanostructured fluid	-	CSGI
Nanorestore Cleaning^®^ Polar Coating G	NPG	Nanostructured fluid	-	CSGI

* NDEK is an aqueous nanostructured fluid containing the solvent diethyl ketone (DExK), NDEC is an aqueous nanostructured fluid containing the solvent diethyl carbonate (DEC), NBuA is an aqueous nanostructured fluid containing the solvent butyl acetate (BuA), NWX is an aqueous nanostructured fluid containing the solvent methyl-tetrahydrofuran (m-THF), and NBA is an aqueous nanostructured fluid containing the solvent benzyl alcohol (BA).

**Table 4:** Hydrogels and organogels used in the tests.

Name	ID	Category	Composition	Provider
Peggy 6	P6	Hydrogel	PVA-based [74]	CSGI
PG PLUS 3	PP3	Hydrogel	PVA-based	CSGI
PVA-SA 2	PSA2	Hydrogel	PVA- and sebacic acid-based [30]	CSGI
PVA-SU 2	PSU2	Hydrogel	PVA- and succinic acid-based [30]	CSGI
PVA-AD	PAD	Hydrogel	PVA- and adipic acid-based [30]	CSGI
CNF-Alginate	CNF-A	Hydrogel	Cellulose nanofibril and alginate biopolymer [31]	UNICAMP
CNF-Gelatin	CNF-G	Hydrogel	Cellulose nanofibril and gelatin biopolymer [31]	UNICAMP
ECO	ECO	Organogel	Poly(hexamethylene diisocyanate), castor oil [39]	CSGI
ECO-Hybrid	ECOH	Organogel	Poly(hexamethylene diisocyanate), castor oil (CO), CO-modified polyethylene glycol (PEG), filler	CSGI
ECO-PEG	ECOP	Organogel	Poly(hexamethylene diisocyanate), castor oil (CO), CO-modified polyethylene glycol (PEG) [39]	CSGI

## Data Availability

Data is contained within the article.

## Abbreviations

The following abbreviations are used in this manuscript:

A	Acetone
ACS	American Chemical Society
A/P	Azelaic/palmiticDirectory of open access journals
AlcTLA	AlcoholsThree letter acronym
AliLD	AliphaticsLinear dichroism
AMDIS	Automated Mass Spectral Deconvolution and Identification System
APAs	Alkylphenyl Alkanoates
Ar	Aromatics
AW	Adjusted water
BA	Benzyl alcohol
C	Chlorinated solvents
CNF-A	Cellulose nanofibrils-alginate biopolymer
CNF-G	Cellulose nanofibrils-gelatin biopolymer
CO	Castor oil
CSGI	Center for Colloid and Surface Science
ECO	Poly(hexamethylene diisocyanate) and castor oil-based organogel named ECO
ECOH	Poly(hexamethylene diisocyanate) and castor oil-based organogel named ECO-Hybrid
ECOP	Poly(hexamethylene diisocyanate) and castor oil-based organogel named ECO-PEG
ESCAPE	Expert System for Characterization using AMDIS Plus Excel
FAMEs	Fatty acid methyl esters
FT-IR	Fourier Transform Infrared spectroscopy
FTIR-ATR	Attenuated Total Reflectance-Fourier Transform Infrared Spectroscopy
GC	Gas chromatography
GCI	Getty Conservation Institute
GC-MS	Gas chromatography-mass spectrometry
G-E	Glycerol ethers and esters
GREENART	GREen ENdeavor in Art Restoration
K	Ketones
LACMA	Los Angeles County Museum of Art
MEK	Methyl ethyl ketone
MSD	Mass selective detector
m-THF	Methyl-tetrahydrofuran
N	Nitrogen containing solvents
NAC	Nanorestore Cleaning^®^ Apolar Coating
NBA	Aqueous nanostructured fluid containing the solvent benzyl alcohol, named HCO BZ2
NBuA	Aqueous nanostructured fluid containing the solvent butyl acetate, named HCO CeXOH-BuAco
NDEC	Aqueous nanostructured fluid containing the solvent diethyl carbonate, named HCO CeXOH-DEC
NDEK	Aqueous nanostructured fluid containing the solvent diethyl ketone, named HCO CeXOH-DEK
NPG	Nanorestore Cleaning^®^ Polar Coating G
NWX	Aqueous nanostructured fluid containing the solvent methyl-tetrahydrofuran, named HCO WX
O/W	Oil-in-water
OM	Optical microscopy
OS	Organic solvent
P/S	Palmitic/stearic
PAD	PVA- and adipic acid-based hydrogel named PVA-AD
PLM	Polarized light microscopy
PP3	PVA-based hydrogel named PG PLUS 3
PSA2	PVA- and sebacic acid-based hydrogel named PVA-SA 2
PSU2	PVA- and succinic acid-based hydrogel named PVA-SU 2
PVA	Poly(vinyl alcohol)
pXRF	Portable X-Ray Fluorescence
RI	Retention index
SEM/EDX	Scanning Electron Microscopy coupled with Energy Dispersive X-Ray Spectroscopy
TEA	Triethanolamine
THM-Py-GC-MS	Pyrolysis-gas chromatography–mass spectrometry with thermally assisted hydrolysis and methylation using tetramethylammonium hydroxide
UN	United Nations
UNICAMP	State University of Campinas
UVF	Ultraviolet fluorescence
VIS	Visible
W	Water
XKA	Hydrogel based on xanthan gum, konjac, and agarose

## Appendix A

### Appendix A.1. Data Related to the pXRF Analysis

**Table A1:** Elements detected at each spot analyzed by pXRF, along with the corresponding instrument settings and analysis mode used. Significant elements are shown in bold, while trace elements are shown in italics.

ID	Color	Instrument	Mode	Elements
1	White	Olympus	Alloy Plus	Fe, Cu, S, Al, Si, Zn, Pb, *Ti*
2	White	Olympus	Precious metal	Fe, Ti, *Pb*, *Cu*
3	White	Olympus	Precious metal	Fe, Pb, *Ti*, *Cr*, *Mn*
4	White	Olympus	Alloy Plus	Fe, Cu, Pb, Al, Si, *Ti*, *Zn*
5	Gold	Olympus	Alloy Plus	Fe, **Cu, Zn**, Pb, Al, Si, S, *Ti*
6	White	Olympus	Alloy Plus	Fe, Al, Si, S, Ti, Cu, *Pb*
7	Gold	Olympus	Alloy Plus	Fe, **Cu, Zn**, Ti, Al Si, S, *Pb*, *Mn*
8	Gold	Olympus	Alloy Plus	Fe, **Cu, Zn**, Pb, Al, Si, *Ti*
9	Silver	Olympus	Alloy Plus	Fe, Ti, Al, Si, Pb, Cu, Zn, S
10	Silver	Olympus	Alloy Plus	Fe, **Cu, Zn**, Pb, Ti, **Al**, Si, S
11	Silver	Olympus	Alloy Plus	**Cu**, *Pb*, *Fe*, *Ti*, *Zn*
12	Gold	Olympus	Alloy Plus	Fe, **Cu,** Ti, **Al,** Pb, **Zn**
13	Orange	Olympus	Geochem	**Pb, Cr**, As, Fe, Ca, Si, Al, *K*
14	Orange	Bruker	Standard	**Pb, Cr,** Fe, Ba, S, *Ca*, *K*, *Cu*, *Ni*
15	Black	Bruker	Standard	Cu, Fe, Ca, Cr, S, Zn, Ti, Si, *Pb*, *Al*
16	Orange	Olympus	Geochem	**Pb,** Fe, As, Al, Si, **S**, Ti, Ca, Cu, K, **Cr, Hg**
17	Black	Olympus	Geochem	Fe, Ca, As, Al, Si, S, K, Pb, *Ti*, *Hg*
18	Black	Bruker	Spectrometer	Pb, Fe, Ca, Cu, K, Hg, *Si*, *Ti*, *Cr*, *Zn*, *Ni*, *Mn*, *Si*
19	Ochre	Bruker	Spectrometer	**Pb,** Fe, Cu, Ca, Ba, **Cr**, Si, *Ti*, *K*, *Ni*, *Al*
20	Red	Bruker	Spectrometer	Pb, Ba, Fe, Cu, Hg, S, Ca, Zn, Si, K, Cr, *Ni*, *Mn*, *Al*
21	Yellow	Bruker	Spectrometer	**Pb, Cr**, Fe, S, Ba, Ca, *Cu*, *Si*
22	Yellow	Bruker	Spectrometer	**Pb, Cr**, Fe, Ca, Ba, Cu, *Si*, *K*, *Ni*, *Al*
23	White	Bruker	Spectrometer	Hg, **Pb**, Fe, Ca, Cu, Ba, Cr, S, *Si*, *K*, *Ti*, *Al*, *Mn*
24	Blue	Bruker	Spectrometer	Hg, Pb, Fe, S, Cu, Ca, Ba, K, Si, Ni, Cr, *Mn*, *Ti*, *Al*
25	Green	Bruker	Spectrometer	**Cu, As**, Hg, *Fe*, *Ti*, *Ca*
26	Green	Bruker	Spectrometer	**Cu, As**, Hg, *Fe*
27	Red	Bruker	Spectrometer	**Hg, S**, Pb, Ba, Fe, Ca, Cu, Cr, Ni, K, Si, *Al*, *Ti*
28	Red	Bruker	Spectrometer	**Hg**, Fe, Pb, **S**, Ca, Cu, K, Ba, Ti, *Mn*, *Si*, *Al*
29	Wood	Bruker	Spectrometer	Pb, Fe, Ca, Cu, Hg, K, Zn, *Ti*, *Mn*, *S*, *Si*, *Al*, *Ba*
30	Wood	Bruker	Spectrometer	Fe, Ca, Cu, Pb, Hg, Zn, K, *Mn*, *Ti*, *Al*
31	Wood	Bruker	Spectrometer	Pb, Fe, Ca, Cu, Hg, K, Mn, *Ti*, *Zn*, *Si*, *Al*
32	Wood	Bruker	Spectrometer	Ca, Fe, Ti, K, Cu, Pb, *Si*, *S*, *Al*

### Appendix A.2. Data Related to the SEM/EDX Analysis

**Table A2:** PLM images in visible light (VIS) and EDX maps of seven cross-sections. Micrographs magnification: 420× (XS1, XS2, XS3, XS4, XS5, XS7), 160× (XS6). EDX maps magnification: 420× (XS1, XS3), 700× (XS2, XS7), 500× (XS4), 550× (XS5), 200× (XS6). XS7 shows only the composition of the ground and the dark layer on the surface.

ID	PLM Images (VIS)	EDX Maps
XS1		
XS2		
XS3		
XS4		
XS5		
XS6		
XS7		

### Appendix A.3. Data Related to the Analysis of the ECOP Loaded in the BA-MO 3:7 Solvent Mixture

**Table A3:** Two-phase exponential decay function used to build the calibration curve described in Section 2.2.2 and Section 4.3.3.

Model	ExpDec2
Equation	y = A1 × exp(−x/t1) + A2 × exp(−x/t2) + y0
Plot	BA/MO signal ratio
y0	−0.08 ± 0.02
A1	3.17 ± 0.26
t1	5.91 ± 0.48
A2	0.62 ± 0.04
t2	43.55 ± 7.24
Reduced Chi-Sqr	1.67 × 10^−5^
R-Square (COD)	1.00
Adj. R-Square	1.00

### Appendix A.4. Areas Treated During the Final Cleaning Tests

**Table A4:** Before- and after-treatment images under visible (VIS) light and ultraviolet fluorescence (UVF), showing the cleaned areas as numbered in Table 2: (1) NBA + Peggy 6; (2) NBA + PP3; (3) NBA + PSA2; (4) NBA + PSU2; (5) NBA + PAD; (6) NBA + CNF-A; (7) NBA + CNF-G; (8) 2% XKA 2:2:1 in DI water + 5% BA; (9) 1% Pemulen™ TR-2 in pH 6.5-AW + 5% BA; (10) ECO + BA-MO 3:7; (11) ECOH + BA-MO 3:7; (12) ECOP + BA-MO 3:7; (13) Evolon^®^ + BA-MO 3:7; (14) BA; (15) BA-MO 3:7. Photos © Yosi Pozeilov, LACMA Conservation Center.

	Before Treatment	After Treatment
VIS		
UVF		

### Appendix A.5. Data Related to the Cleaning Tests

**Table A5:** Micrographs acquired with the digital microscope (HIROX) in visible light (VIS). Micrographs magnification: 100×. Cleaned areas as numbered in Table 2: (1) NBA + Peggy 6; (2) NBA + PP3; (3) NBA + PSA2; (4) NBA + PSU2; (5) NBA + PAD; (6) NBA + CNF-A; (7) NBA + CNF-G; (8) 2% XKA 2:2:1 in DI water + 5% BA; (9) 1% Pemulen™ TR-2 in pH 6.5-AW + 5% BA; (10) ECO + BA-MO 3:7; (11) ECOH + BA-MO 3:7; (12) ECOP + BA-MO 3:7; (13) Evolon^®^ + BA-MO 3:7; (14) BA; (15) BA-MO 3:7.

**Spot**	**Before Treatment**	**After Treatment**
1		
2		
3		
4		
5		
6		
7		
8		
9		
10		
11		
12		
13		
14		
15		
